# Structural changes in the transport cycle of the mitochondrial ADP/ATP carrier

**DOI:** 10.1016/j.sbi.2019.03.029

**Published:** 2019-08

**Authors:** Jonathan J Ruprecht, Edmund RS Kunji

**Affiliations:** MRC Mitochondrial Biology Unit, University of Cambridge, Cambridge Biomedical Campus, Cambridge, CB2 0XY, UK

## Abstract

The mitochondrial ADP/ATP carrier, also called adenine nucleotide translocase, accomplishes one of the most important transport activities in eukaryotic cells, importing ADP into the mitochondrial matrix for ATP synthesis, and exporting ATP to fuel cellular activities. In the transport cycle, the carrier changes between a cytoplasmic and matrix state, in which the central substrate binding site is alternately accessible to these compartments. A structure of a cytoplasmic state was known, but recently, a structure of a matrix-state in complex with bongkrekic acid was solved. Comparison of the two states explains the function of highly conserved sequence features and reveals that the transport mechanism is unique, involving the coordinated movement of six dynamic elements around a central translocation pathway.

## Highlights

• First structure of the matrix-open state of a mitochondrial ADP/ATP carrier has been solved.
• Structure determined using protein inhibited by the toxin bongkrekic acid, which blocks the substrate-binding site.
• Structural comparison shows highly dynamic conformational changes during transport with six mobile elements.
• Proposed mechanism explains roles of all conserved sequence features in mitochondrial carriers.

## Graphical abstract

**Figure:**
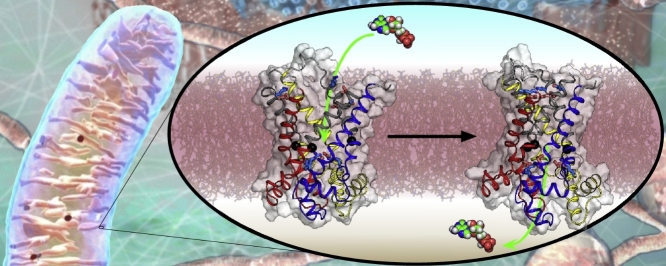

**Current Opinion in Structural Biology** 2019, **57**:135–144

This review comes from a themed issue on **Membranes**

Edited by **Jonathan Ruprecht** and **Edmund Kunji**

For a complete overview see the Issue and the Editorial

Available online 28th April 2019

**https://doi.org/10.1016/j.sbi.2019.03.029**

0959-440X/© 2019 The Authors. Published by Elsevier Ltd. This is an open access article under the CC BY license (http://creativecommons.org/licenses/by/4.0/).

## Introduction

**Figure 1:**
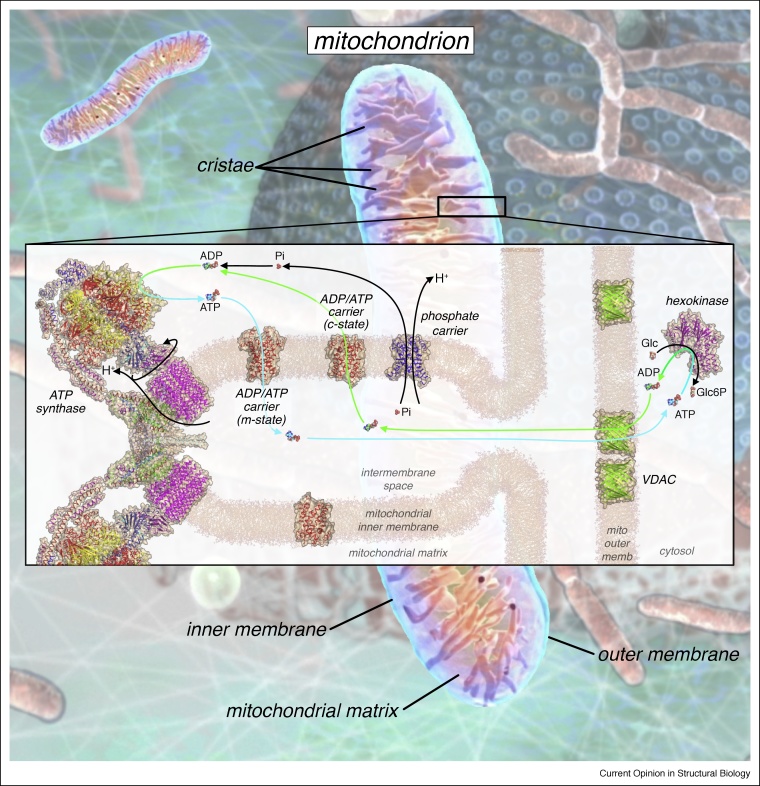
The role of ADP and ATP transport in oxidative phosphorylation. In eukaryotes, the mitochondrion is the site of oxidative phosphorylation. The impermeable mitochondrial inner membrane forms characteristic invaginations, called cristae, and contains the respiratory complexes (not shown), ATP synthase, and transport proteins including the ADP/ATP carrier and phosphate carrier. ATP drives many biochemical reactions (exemplified by hexokinase, PDB: 3B8A, converting glucose to glucose-6-phosphate). The spent fuel ADP crosses the mitochondrial outer membrane via voltage-dependent anion channels (VDAC, PDB: 3EMN) and diffuses into the mitochondrial intermembrane space. ADP binds to mitochondrial ADP/ATP carriers in the c-state (PDB: 4C9H chain A), inducing a conformational change that switches the carrier to the m-state, leading to transport of ADP across the membrane. Phosphate ions are transported across the mitochondrial inner membrane by the phosphate carrier (homology model based upon PDB: 4C9H chain A), in symport with protons. ADP and phosphate are the substrates for ATP synthase (shown in its dimeric form at the sharply curved edge of the crista, PDB: 6B8H). ATP synthase uses the proton motive force generated by the respiratory complexes to drive the rotary mechanism of ATP synthesis [49]. The product, ATP, binds to mitochondrial ADP/ATP carriers in the m-state (PDB: 6GCI chain A), driving a conformational change to the c-state and transport of ATP into the intermembrane space, from where it can diffuse to the cytosol and other cell organelles to fuel more reactions.

The mitochondrial ADP/ATP carrier performs the vital task of importing ADP into the mitochondrial matrix, where it can be converted into ATP by ATP synthase, and exporting the newly synthesized ATP to the cytosol, where it fuels metabolic energy-requiring processes of the cell [1^••^]. It has been estimated that human ADP/ATP carriers transport approximately 65 kg of ATP across the mitochondrial inner membrane every day to sustain our activities [2,3], meaning that on average each ATP molecule is used and synthesized again every minute (Figure 1). The carrier is the archetypal member of the mitochondrial carrier family, which is the largest transporter family in humans with 53 members. They play key roles in transporting metabolites, inorganic ions, and cofactors across the mitochondrial inner membrane, linking metabolic pathways in the mitochondrial matrix to the cytosol, transporting building blocks for maintenance, and exchanging ions and compounds for homeostasis [4,5^•^,^6^].

The ADP/ATP carrier is relatively small (≈30 kDa), yet its substrates are among the largest and most highly charged solutes to cross biological membranes. Transport of adenine nucleotides is achieved without inducing significant proton leak across the mitochondrial inner membrane, which would be detrimental to the energy provision of the cell. How the protein achieves these feats has been of interest since the protein was first identified in the early 1960s [7, 8, 9]. The carrier catalyses the equimolar exchange of ADP and ATP across the inner mitochondrial membrane [10, 11, 12]. *In vitro*, the carrier can transport either nucleotide in either direction across the membrane [13,14]. *In vivo*, the directionality is determined by the concentration gradient of adenine nucleotides, and by the membrane potential, since the exchange of ADP and ATP is electrogenic [13, 14, 15, 16]. Two-specific inhibitors are available, and have greatly facilitated biochemical and biophysical studies of the carrier. Atractylosides, such as atractyloside (ATR) or carboxyatractyloside (CATR), lock the carrier in a cytoplasmic open state (c-state) in which the central substrate-binding site is accessible to the intermembrane space, which is confluent with the cytosol (Figure 1) [17, 18, 19]. Bongkrekic acid (BKA), and the related isobongkrekic acid, lock the carrier in a matrix open state (m-state) with the central binding site accessible to the mitochondrial matrix [20,21].

**Figure 2:**
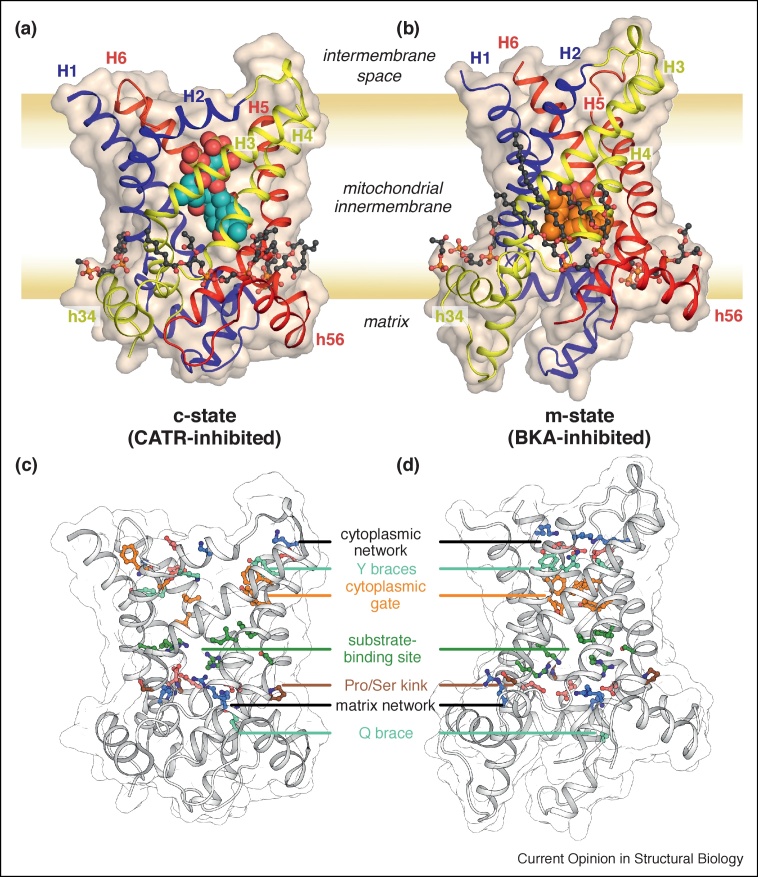
Structure of fungal mitochondrial ADP/ATP carriers. **(a)** The c-state (inhibited by CATR, PDB: 4C9H chain A) and **(b)** the m-state (inhibited by BKA, PDB: 6GCI chain A). The carrier proteins are shown in cartoon representation, colored by domain (blue, domain 1; yellow, domain 2; red, domain 3), and as a wheat-colored surface. Transmembrane α-helices (H1–H6) and matrix α-helices (h34 and h56, h12 is not labelled) are indicated. The inhibitors are shown in sphere representation with cyan carbons for CATR and orange carbons for BKA. **(c)** and **(d)**, the same views of the two states, respectively, but with highly conserved sequence features highlighted in color, as indicated.

The complete amino acid sequence of the bovine ADP/ATP carrier provided the first sequence information for any member of the mitochondrial carrier family [22]. The sequence contained a striking pattern of three homologous sequence repeats, each about 100 amino acids long, predicting that the carrier would have six transmembrane α-helices [23]. The first structural information was obtained for the yeast ADP/ATP carrier Aac3p in complex with ATR by electron crystallography, demonstrating that the carrier was structurally monomeric and threefold pseudo-symmetrical with a central translocation pathway for adenine nucleotides [24]. The structure of the bovine ADP/ATP carrier (PDB: 1OKC and 2C3E), trapped in the c-state by CATR, was one of the first transport proteins to be determined at atomic resolution [25^••^,^26^]. It showed that the carrier is composed of three domains, each comprising an odd-numbered transmembrane α-helix (H1, H3, or H5), a loop with a short matrix α-helix (h12, h34, or h56) lying in the plane of the membrane, and an even-numbered transmembrane α-helix (H2, H4, or H6). The basic structural fold was confirmed later by the structures of the yeast isoforms Aac2p (PDB: 4C9G and 4C9H) and Aac3p (PDB: 4C9J and 4C9Q) (Figure 2(a) and (c)) [27^•^]. The odd-numbered α-helices have pronounced kinks, located at the proline or serine residues of a highly conserved [PS]x[DE]xx[KR] motif, giving them an L-shape [25^••^,^26^,27^•^]. The charged residues of these motifs on the odd-numbered helices form inter-domain salt-bridges [25^••^,^26^,27^•^,^28^], now called the matrix salt-bridge network [29^•^], which helps to close access to the cavity from the matrix side when the protein is in the c-state. The salt bridge residues can be braced by glutamine residues, which provide additional inter-domain interactions stabilizing the matrix network in the c-state [27^•^]. CATR inhibits the protein by binding to the central cavity, blocking the translocation pathway and forming multiple salt-bridges and hydrogen-bonds with the carrier [25^••^,27^•^], explaining its ability to increase the thermal stability of the carrier significantly [30].

In the absence of an m-state structure, bioinformatic and biophysical methods have helped to identify key functional elements that are important for the transport mechanism. In particular, a single central substrate-binding site was identified in all mitochondrial carriers using chemical and distance restraints [31,32] and symmetry analysis [29^•^]. Molecular dynamics simulations have shown that ADP binds to this site in the ADP/ATP carrier [12,33,34]. Furthermore, the charged residues of a highly conserved [YF][DE]xx[KR] motif on the even-numbered α-helices were proposed to form inter-domain salt-bridges in the m-state, now called the cytoplasmic salt-bridge network [29^•^]. The importance of this network has been confirmed by analysis of the transport properties [27^•^,35^•^], thermostability [35^•^] and energetics [36^•^] of the carrier. It has been proposed that substrate binding drives the interconversion between the c-state and m-state by disruption and formation of the matrix and cytoplasmic salt-bridge networks, making the central substrate-binding site alternately accessible to either side of the membrane [1^••^,27^•^,29^•^].

Whereas the structures of the CATR-inhibited ADP/ATP carriers provided significant insight into the c-state, it was not possible to infer the conformational changes that the carrier undergoes when it transitions to the m-state. Recent advances have led to the first structure determination of a mitochondrial ADP/ATP carrier in an m-state [37^••^]. This review summarizes the key features of the structure, and explains the new insights into the transport mechanism.

## Purification and crystallization of an ADP/ATP carrier trapped in the m-state

The low stability of the m-state in detergents made its structure determination extremely challenging [30]. The first step was the expression and purification of an N-terminal His-tagged ADP/ATP carrier from the thermotolerant fungus *Thermothelomyces thermophila* (TtAac) [30]. TtAac has 75% sequence identity to ScAac2 and ScAac3, and 51% identity to the bovine ADP/ATP carrier. The carrier was expressed in *Saccharomyces cerevisiae* strain WB-12, which lacks endogenous functional ADP/ATP carriers and cannot grow on non-fermentable carbon sources [38]. Expression of the carrier complemented the growth defect, showing that significant amounts of functional carrier were being produced. It was possible to purify the protein in the m-state by using the state-specific inhibitor BKA and maltose–neopentyl glycol detergents, which improve the stability of the detergent-solubilized protein. Small crystals could be grown using standard vapor-diffusion techniques, but the crystals were unstable, dissolving within days in the crystallization drops. Fungal ADP/ATP carriers are unusual in having an atypical glutamine (Q302) instead of a lysine residue on the C-terminal end of H6, producing a weaker cytoplasmic salt-bridge network compared to human and bovine carriers [35^•^]. Consistent with this, the Q302K mutation of TtAac, which mimics the network residues found in human and bovine ADP/ATP carriers, shows an increased thermal stability of the BKA-inhibited protein [35^•^]. Using this mutant, it was possible to produce crystals of the BKA-inhibited protein that persisted for longer within the crystallization drops. Unfortunately, most crystals diffracted poorly, with only one crystal out of many hundreds diffracting anisotropically to 3.6 Å resolution in the best direction [37^••^]. To improve the diffraction quality of the crystals and to facilitate structure determination, nanobodies were raised against BKA-inhibited TtAac which had been reconstituted into proteoliposomes. Nanobodies are single-domain antibody fragments produced by camelids [39], which have the structural and functional properties of the heavy-chain only antibodies, are easy to express in *Escherichia coli*, and have proven to be excellent crystallization chaperones for membrane proteins [40]. State-specific nanobodies were selected that bound specifically to the BKA-inhibited state, but not to the CATR-inhibited state of TtAac. By adding a Factor Xa-cleavable His tag to the nanobodies, it was possible to purify BKA-inhibited untagged TtAac from mitochondrial membranes, and explore a wider range of detergents for crystallization. The detergent decanoyl-*N*-hydroxyethylglucamide (HEGA-10) was particularly useful, producing crystals that routinely diffracted anisotropically to beyond 4-Å resolution. The crystal used for structure determination diffracted to beyond 3.3-Å resolution in the best direction [37^••^]. It was possible to determine phases by molecular replacement by placing a model of the nanobody, followed by the sequential addition of fragments of the domains of yeast Aac2p (PDB: 4C9H chain A). As refinement progressed, densities for loops, BKA and cardiolipin appeared, showing the validity of the phasing solution.

## Overall structure of the BKA-inhibited m-state

The structure of the BKA-inhibited m-state shows six transmembrane α-helices surrounding a central cavity, which is open to the mitochondrial matrix (Figure 2(b) and (d)). The characteristic three domain structure of the family is apparent, with the matrix helices still lying parallel to the membrane, but rotated outwards compared to the c-state, helping to open the central cavity to the matrix. Residues of the matrix salt-bridge network (E37, K40, D142, R145, D242 and R245) and cytoplasmic salt-bridge network (D101, K104, D205, K208, D299 and Q302K) flank the central substrate-binding site (residues K30, R88, G192, I193, Y196, S238 and R287) of TtAac. Surface representations reveal the dramatic difference in shape of the protein in the c-state and m-state (Figure 2). The structure shows the inhibitor BKA blocking the central substrate translocation pathway, and the positions of associated cardiolipin lipid molecules.

## Substrate-binding site

Both c-state and m-state structures show that the predicted residues of the substrate-binding site [29^•^,^31^,^32^] lie approximately in the middle of the membrane. The phosphate moieties of adenine nucleotides most likely bind to residues K30, R88, and R287, with the adenine moiety bound in a pocket consisting of residues G192, I193, Y196, and S238 of TtAac. When ADP is bound to this site, it is neutralized, facilitating import of ADP against the negative inside membrane potential, but when ATP is bound, a net minus charge remains, facilitating its export in the direction of the positive outside membrane potential. The binding site residues are alternately accessible to either side of the mitochondrial inner membrane as the carrier switches between c-state and m-state (Figure 2) which holds for many transport proteins [41]. This notion is in agreement with the single-binding center gated pore hypothesis [42], which was based on a careful analysis of the two types of inhibitors. While there are subtle differences in the geometry of the substrate-binding site residues and their local chemical environment, the same residues are accessible from either side of the membrane in each state. Combined with the chemical similarity of ADP and ATP, this is consistent with the known equimolar exchange activity of the carrier, and with the fact that either nucleotide can be transported in either direction *in vitro*.

**Figure 3:**
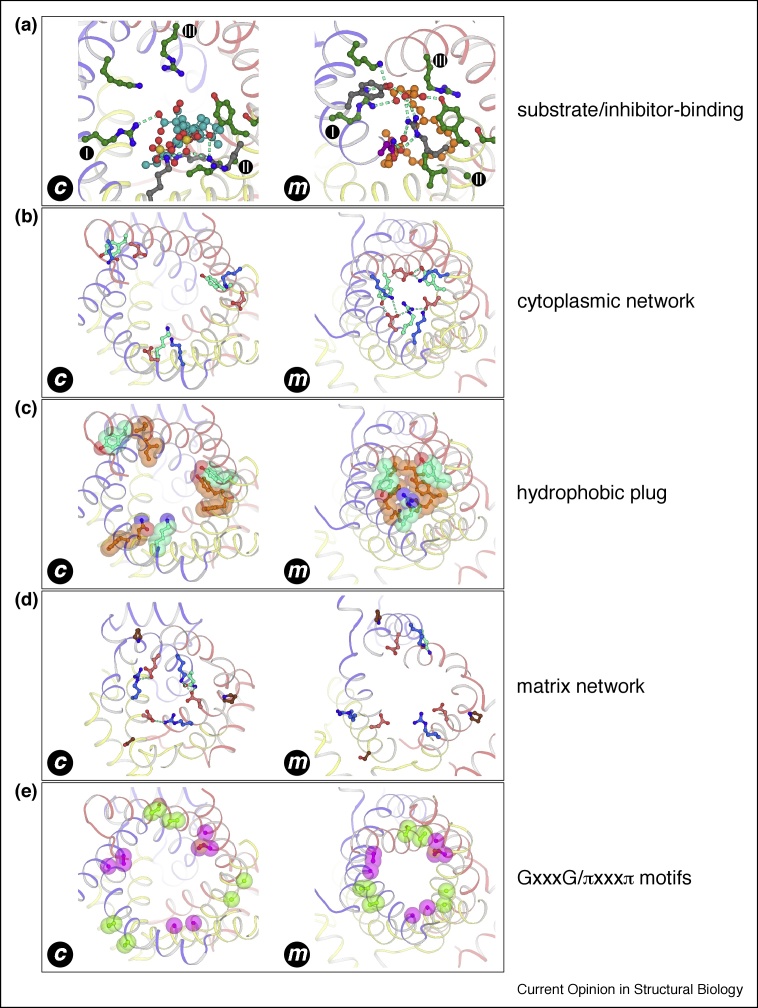
Key structural elements required for the transport mechanism, observed in the c-state and m-state structures. Domains are represented and colored as in Figure 2. **(a)** Substrate-binding site residues, colored in green. Also shown are the bound inhibitors CATR (cyan carbons) and BKA (orange carbons). **(b)** The cytoplasmic network, showing positively charged (blue carbons) and negatively charged residues (red carbons). The tyrosine braces and arginine substitution are shown with cyan carbons. **(c)** Residues of the hydrophobic plug (orange carbons), which includes the tyrosine brace (cyan carbons). **(d)** The matrix network, with charged residues shown as in (b). The glutamine brace is shown with cyan carbons. The proline/serine kink residues are shown in brown. BKA has been removed from the view of the m-state structure, for clarity, and E37 has been modelled as a common rotamer. **(e)** Residues of the GxxxG (magenta carbons) and πxxxπ motifs (green carbons).

In both c-state and m-state structures, the inhibitor sits within the central cavity and interacts directly with residues of the substrate-binding site (Figure 3(a)), demonstrating that both are competitive inhibitors. They form multiple salt-bridges and hydrogen bonds to protein residues, explaining their known tight-binding. Bongkrekic acid adopts an unusual horseshoe shape, facilitated by its polyunsaturated backbone, which allows the carboxylate groups at either end of the molecule to maximize their interactions with nearby residues, including those of the substrate-binding site.

## Salt bridge networks and gates in the c-state and m-state

The BKA-inhibited m-state structure provides the first direct visualization of the formation of the cytoplasmic salt-bridge network in the m-state (Figure 3(b)). In the m-state, the even-numbered transmembrane α-helices come close together towards the cytoplasmic side of the membrane, enabling the charged residues of the [YF][DE]xx[KR] motif to interact. The structure also reveals that tyrosine residues of the motif provide additional inter-domain hydrogen bonds to the negatively charged residues of the motif, bracing the salt-bridge network, in agreement with theoretical predictions [36^•^]. These ‘tyrosine brace’ residues are not absolutely conserved across the mitochondrial carrier family; hence the cytoplasmic network strength can vary. ADP/ATP carriers are unusual members of the mitochondrial carrier family, since an arginine residue replaces the tyrosine of the [YF][DE]xx[KR] motif on H2. Nevertheless, the arginine provides an additional inter-domain interaction for the cytoplasmic network, mimicking the role of tyrosine. Below the tyrosine brace, additional aromatic or bulky hydrophobic residues are found (e.g. N96, F97, Y200, F201, V294, L295), which block access to the substrate-binding site from the cytoplasmic side of the membrane. Together with the cytoplasmic salt-bridge network, these residues constitute the cytoplasmic gate, which provides a thick (≈15 Å) hydrophobic plug (Figure 3(c)). Since the central cavity is open to the matrix-side of the membrane, the residues of the matrix salt-bridge network lie far apart and are not interacting (Figure 3(d)).

The CATR-inhibited c-state structures show the converse set of network interactions compared to the m-state. The residues of the cytoplasmic salt-bridge network and hydrophobic plug lie far apart, as the carrier is open to the cytoplasmic side of the membrane (Figure 3(b) and (c)). The residues of the matrix salt-bridge network, however, are engaged (Figure 3(d)). The odd-numbered transmembrane α-helices lie close together towards the matrix side of the membrane, allowing the charged residues of the [PS]x[DE]xx[KR] motifs to form salt-bridge interactions, forming the matrix salt-bridge network [25^••^,^26^,27^•^]. The matrix salt-bridge network is braced by a glutamine residue [27^•^], providing additional inter-domain interactions that help to stabilize the c-state. The matrix salt-bridge network, together with the C-terminal ends of the odd-numbered transmembrane α-helices, form the matrix gate, which blocks access to the central cavity from the matrix side of the membrane. In the fungal ADP/ATP carriers, an additional turn of α-helix at the C-terminal end of H1, which is a loop in the bovine carrier, sits directly below the central pseudosymmetry axis, contributing to the gate [27^•^]. The gates prevent proton leak across the mitochondrial inner membrane in both states, and control access to the central cavity and substrate-binding site.

The structures demonstrate that formation and disruption of the cytoplasmic and matrix network are key aspects of the transport mechanism, as predicted [27^•^,29^•^,35^•^]. In each mitochondrial carrier, the strength of the networks is modulated by different numbers of bonds and braces, which are likely to be adaptations to allow different modes of transport, that is, strict equimolar exchange versus uniport [29^•^,36^•^].

## Inter-helical interfaces: role of the GxxxG and πxxxπ motifs

A striking feature of the BKA-inhibited m-state structure is the close packing of transmembrane α-helices on the cytoplasmic side of the membrane (Figure 2(b) and (d)), which is required for residues of the cytoplasmic network to bond. The close packing is facilitated by amino acids with small side chains lying at the intra-domain interface between odd-numbered and even-numbered transmembrane α-helices. On the odd-numbered α-helices they include the residues of the GxxxG motif, highly conserved across the mitochondrial carrier family (Figure 3(e)). On the even-numbered α-helices, they consist of amino acids with small side chains, which form the πxxxπ motif (π being the one letter code for small side chain amino acids) (Figure 3(e)). Residues of the GxxxG and πxxxπ motifs lie far apart in the c-state structures, and thus it was not possible to explain the conservation of these residues in the absence of an m-state structure.

## Conformational changes between c-state and m-state

**Figure 4:**
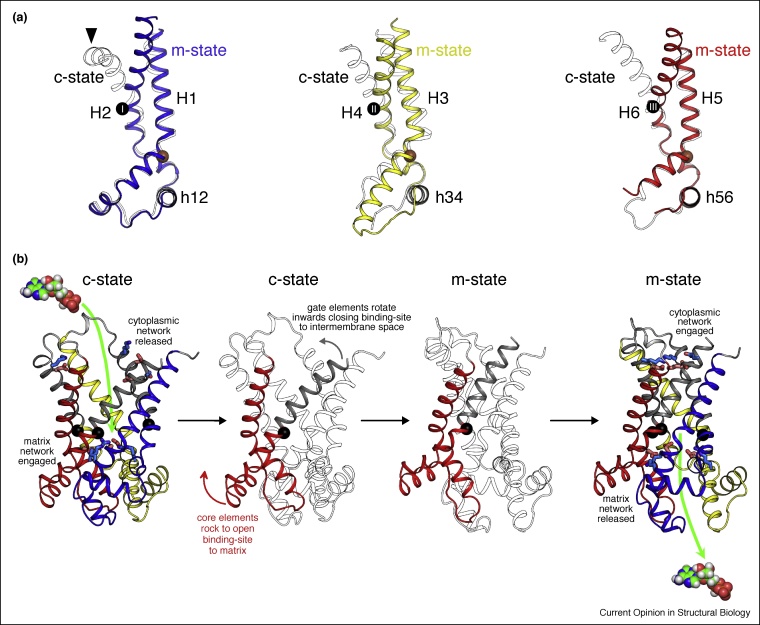
Proposed transport mechanism of the mitochondrial ADP/ATP carrier. **(a)** Conformational changes between c-state (PDB: 4C9H chain A, shown in outline) and m-state (PDB: 6GCI chain A) shown for each domain of the carrier. For the m-state, domains 1–3 are shown as blue, yellow, and red cartoons, respectively. The orange spheres mark the positions of the conserved prolines/serines of the [PS]x[DE]xx[KR] motif. The numbered black spheres mark the positions of the substrate-binding site contact points. Domains have been aligned on their core elements. **(b)** Conformational changes between modelled uninhibited c-state and m-state of the ADP/ATP carrier, viewed laterally from the membrane. Core elements 1, 2, and 3 are colored by domain in blue, yellow, and red, respectively, and the gate elements are colored grey. Conformational changes between c-state and m-state can be described as a rotation of the core elements, coupled with an inward movement of the gate elements, and are highlighted in the middle panels for domain 3.

It became apparent early on during model building of the m-state that the structures of the individual carrier domains were not exactly conserved between c-state and m-state (Figure 4(a)). While most of the domain structure is conserved between the states, there is a significant change in the position of the C-terminal regions of the even-numbered α-helices. Each domain is, therefore, composed of two elements which move differently during the transition between c-state and m-state. The conserved bulk of the domains are now called the core elements, whereas the C-terminal ends of the even-numbered α-helices are named the gate elements. For each domain, the junction between core and gate elements occurs at key substrate-binding site residues (R88, G192 and R287 in TtAac), which form the contact-points of the binding site [31,32]. This analysis provides key information about the nature of the conformational changes. The transition between c-state and m-state must involve a movement of the three core elements as a rigid-body, opening up access to the substrate-binding site from the matrix side of the membrane, and a concomitant inward movement of the gate elements towards the central axis of the carrier. Movement of the gate elements is essential for the formation of the cytoplasmic gate and network, closing access to the substrate-binding site from the cytoplasmic side of the membrane. Previous proposals that the proline residues of the signature motifs act as flexible hinges, allowing the odd-numbered helices to straighten out during transport [25^••^] or that proline and glycine residues act as hinges of six mobile elements per domain [43] have now been proven to be incorrect.

## From inhibited states to a model of the transport mechanism

How do the observed domain changes operate in the context of the carrier? A simple morph between c-state and m-state structures does not provide a satisfactory answer, because clefts open up between helices in the lipid-facing part of the structure, which is energetically unlikely, and there is no occluded intermediate state, in which access to the substrate-binding site is blocked from both sides of the membrane. An occluded intermediate state is required to prevent proton leak across the mitochondrial inner membrane. The likely reason for these problems with the morph is that the inhibitors have distorted parts of each structure. CATR and BKA both have larger molecular volumes than ADP or ATP, and they form multiple polar interactions with this highly dynamic protein. For these reasons, it has been suggested that the inhibitors induce structural changes to the protein, thus maximising available interactions and their binding affinity, while producing abortive states [44]. Analysis of the CATR-inhibited and BKA-inhibited structures supports this notion [37^••^]. CATR appears to induce a kink at the C-terminal region of H2 (black arrow head in Figure 4(a)), which in turn induces a movement of the N-terminal region of H3. BKA causes a rigid-body displacement of domain 1 of the carrier, pushing it outwards on the matrix side of the membrane, where there is a noticeable deviation from the threefold pseudosymmetry.

Nevertheless, by comparing the inhibited structures, it has been possible to generate models of the uninhibited c-state and m-state of the carrier [37^••^]. A morph between the uninhibited models does provide a plausible transport mechanism, via an occluded intermediate state. The mechanism involves six mobile elements, with the core elements of each domain rocking outwards during the transition from c-state to m-state, opening up the substrate-binding site to the matrix side of the membrane (Figure 4(b)). Simultaneously, the gate elements rotate inwards, closing the binding site to the intermembrane space and cytosol. The transition from m-state to c-state involves the same elements operating in reverse. The contact points of the substrate-binding site act as the pivot points for the conformational changes, linking substrate binding to the induction of conformational changes. The involvement of three contact points in substrate binding, one on each domain, may help to ensure that the movements of the gate and core elements are simultaneous and concurrent in all three domains. In the m-state in which the matrix salt bridge network is disrupted, the cardiolipin molecules form key inter-domain interactions that hold the carrier together on the matrix side. This analysis shows that the mitochondrial ADP/ATP carrier has a unique transport mechanism, and is the most dynamic transporter characterized to date.

## Future directions

The BKA-inhibited structure has revealed many of the key structural features of the m-state, has explained the role of previously unexplained conserved sequence motifs, and has revealed the molecular basis for the conformational changes between c-state and m-state. There are still important questions to answer. In particular, how does substrate-binding drive the conformational changes? Insight into this process may come from biophysical, computational and structural studies of the substrate-bound states. These structures may also help our understanding of transport specificity — why is it that ADP and ATP can be transported, but not GDP or GTP [12,45]? It is likely that the mechanism applies across the mitochondrial carrier family of proteins, but substrate sizes vary quite widely from a single proton to Coenzyme A. The aspartate-glutamate and ATP-Mg/Pi carriers contain additional calcium-regulated domains. While the structures of regulatory domains from both proteins are available [46, 47, 48], further work is required to understand how calcium-binding can control the transport steps by the carrier domains. Further work is also required to understand how mutations cause dysfunctional mitochondrial carriers, which in turn lead to neuromuscular, metabolic and developmental diseases.

## Conflict of interest statement

Nothing declared.

## References and recommended reading

Papers of particular interest, published within the period of review, have been highlighted as:

• of special interest
•• of outstanding interest
